# Barriers and Facilitators to the Implementation of a Mobile Insulin Titration Intervention for Patients With Uncontrolled Diabetes: A Qualitative Analysis

**DOI:** 10.2196/13906

**Published:** 2019-07-31

**Authors:** Erin Rogers, Sneha R Aidasani, Rebecca Friedes, Lu Hu, Aisha T Langford, Dana N Moloney, Natasha Orzeck-Byrnes, Mary Ann Sevick, Natalie Levy

**Affiliations:** 1 Department of Population Health New York University School of Medicine New York, NY United States; 2 Department of Medicine New York University School of Medicine New York, NY United States

**Keywords:** type 2 diabetes, telemedicine, implementation science

## Abstract

**Background:**

In 2016, a short message service text messaging intervention to titrate insulin in patients with uncontrolled type 2 diabetes was implemented at two health care facilities in New York City.

**Objective:**

This study aimed to conduct a qualitative evaluation assessing barriers to and the facilitators of the implementation of the Mobile Insulin Titration Intervention (MITI) program into usual care.

**Methods:**

We conducted in-depth interviews with 36 patients enrolled in the MITI program and the staff involved in MITI (n=19) in the two health care systems. Interviews were transcribed and iteratively coded by two study investigators, both inductively and deductively using a codebook guided by the Consolidated Framework for Implementation Research.

**Results:**

Multiple facilitator themes emerged: (1) MITI had strong relative advantages to in-person titration, including its convenience and time-saving design, (2) the free cost of MITI was important to the patients, (3) MITI was easy to use and the patients were confident in their ability to use it, (4) MITI was compatible with the patients’ home routines and clinic workflow, (5) the patients and staff perceived MITI to have value beyond insulin titration by reminding and motivating the patients to engage in healthy behaviors and providing a source of patient support, and (6) implementation in clinics was made easy by having a strong implementation climate, communication networks to spread information about MITI, and a strong program champion. The barriers identified included the following: (1) language limitations, (2) initial nurse concerns about the scope of practice changes required to deliver MITI, (3) initial provider knowledge gaps about the program, and (4) provider perceptions that MITI might not be appropriate for some patients (eg, older or not tech-savvy). There was also a theme that emerged during the patient and staff interviews of an unmet need for long-term additional diabetes management support among this population, specifically diet, nutrition, and exercise support.

**Conclusions:**

The patients and staff were overwhelmingly supportive of MITI and believed that it had many benefits and that it was compatible with the clinic workflow and patients’ lives. Initial implementation efforts should address staff training and nurse concerns. Future research should explore options for integrating additional diabetes support for patients.

## Introduction

Over 30 million people in the United States have diabetes [1], and approximately 30% of adults with diabetes take insulin to regulate their blood sugar [2]. The process of finding the correct dose of insulin often requires frequent visits to a clinician to review daily fasting blood glucose (FBG) logs and adjust doses until the patient reaches a desired FBG level. This insulin titration process is demanding for both patients and health care systems. For patients, it requires daily FBG testing and tracking. Patients must also devote considerable time and effort to make medical appointments, potentially contributing to lost wages or absence in school and extra childcare costs [3,4]. For health care systems, it requires the availability of appointments and clinician time for titration visits. This can be especially challenging for public and safety net hospitals that care for large numbers of patients with diabetes [5].

Mobile health (or mHealth) interventions have been promoted as a means to overcome barriers to health care in general and safety net populations. These interventions use mobile devices, such as mobile phones, to provide patient education and care support in between health care visits. Through the use of phone calls, text messaging, and/or smartphone apps, mHealth interventions may alleviate the barriers to health care access by eliminating costs of commuting to medical visits, difficulties finding childcare to attend medical visits, difficulties taking time off for health care visits, and limits in appointment availability. Previous studies have supported the effectiveness of mHealth interventions for diabetes self-management [6]; however, there are no mobile programs to assist patients with type 2 diabetes with the process of insulin titration.

In recognizing the power of mHealth to help vulnerable populations access care, Bellevue Hospital (part of NYC Health + Hospitals in New York City) developed a remote insulin titration program called the *Mobile Insulin Titration Intervention* (MITI). With MITI, an automated system sends a text message every week day to patients who have uncontrolled type 2 diabetes and need an adjustment of their basal insulin by asking for their FBG levels. A nurse monitors incoming FBG texts from patients, and nurses call patients once weekly to advise on insulin dose titration. The hospital also supplies uninsured patients with free FBG testing supplies. The goal of MITI is to find the dose of insulin that achieves an FBG level of 80 to 130 mg/dL [7]. In a randomized controlled trial (RCT), 88% (29/33) of MITI patients (vs 37% [10/27] of patients receiving the usual care) were able to find their optimal insulin dose (OID) in an average of 3 weeks [8].

On the basis of the success of the RCT, Bellevue Hospital and a second NYC Health + Hospitals facility (Gouverneur Health) implemented the MITI program into routine care. To accelerate the future translation of MITI in other settings, we conducted a mixed-methods hybrid effectiveness implementation study evaluating the implementation of MITI as it moved from the RCT into usual care [9,10]. The study’s quantitative analysis showed that MITI was effective as routine care, with 84% of MITI patients reaching their OID in an average of 24 days [9]. This paper describes the study’s qualitative analysis assessing barriers and facilitators to implementing MITI into routine care.

## Methods

### Setting

The study took place at two ambulatory care facilities within NYC Health + Hospitals: Bellevue Hospital and Gouverneur Health. Bellevue is the nation’s oldest public hospital, providing over 60,000 primary care continuity visits per year to 33,000 active patients. Gouverneur Health is NYC Health + Hospitals’ largest ambulatory care facility, providing over 267,000 outpatient visits per year. Each site cares for approximately 5000 patients with diabetes annually and serves a multiethnic, multiracial patient population. Most patients (65% at Bellevue and 75% at Gouverneur) either have Medicaid or are uninsured. This study was approved by the New York University institutional review board (IRB).

### Routine Insulin Titration Care

The patients with diabetes needing titration of basal insulin have the option of self-titration [11]. The patients who are not comfortable with self-titration are asked to keep a daily log of their FBG level and return to the clinic to review the FBG log in person with a provider to identify the need for a change in their insulin dose. The frequency and the timing of in-person visits to check the FBG logs can vary based on patient needs.

### Mobile Insulin Titration Intervention Program

Physicians could refer patients with uncontrolled diabetes who needed adjustment of their basal insulin to MITI during a routine clinical visit (full patient eligibility criteria are described elsewhere [10]). At Bellevue, physicians referred the patients to MITI by paging an onsite program coordinator who met the patient to complete enrollment. At Gouverneur, physicians referred the patients to their regular team nurse who completed enrollment as part of the routine outtake process. Each week day, enrolled patients received a text message from a secure Web portal [12] asking “What was your fasting blood sugar this morning?”, and patients texted back the value. Every week day, a nurse monitored incoming values for alarming values or anomalies. If a patient texted on a weekend or afterhours, he or she received an automated text message stating the following:

There is no one available to review this text at this time. Your message will be reviewed on the next day, usually from Monday to Friday.

Every Thursday afternoon a nurse would call each MITI patient to provide dosing instructions using an algorithm [8,10] developed by the MITI clinical director (NL). At Bellevue, the daily FBG monitoring and Thursday titration calls were completed by the clinic’s two diabetes nurse educators (DNEs) who had protected time to deliver the MITI intervention. At Gouverneur, these procedures were performed by the team nurses who enrolled the patient in MITI without protected time. Patients remained in MITI for up to 12 weeks and were discharged when they (1) achieved their OID either by reaching an FBG level within 80 to 130 mg/dL or by reaching the maximum dose of 50 units, (2) had to be terminated early because a nurse was unable to reach them by phone after 3 consecutive weeks to provide titration instructions, or (3) had been in the program for 12 weeks without reaching their OID.

### Implementation Process

The MITI team used a combination of evidence-based strategies to implement MITI at each site [13]. Before implementation, an advisory committee was formed at each site comprising physician and nursing representatives who helped decide how MITI would function as routine care in their clinic (eg, staffing model and referral process). Once MITI was ready to start, the team disseminated educational materials and conducted educational activities with providers and nurses. These activities included group and individual training sessions with the nurses delivering MITI on how to enroll, monitor, and titrate patients. The team also presented the program and distributed MITI materials to physicians at regular staff meetings. After implementation, the MITI team met with physicians and nurses at routine staff meetings to provide program updates and hear clinician feedback. The MITI team performed an individual educational outreach to new physicians and nurses as needed. The MITI coordinator was also available to assist the nurses if they were having challenges with the texting platform or other program elements.

### Interview Participants and Recruitment

#### Patients

We were interested in interviewing 50 patients enrolled in MITI (25 per site). During our interview recruitment period, all patients enrolled in the MITI program as part of their routine care were invited to participate in an interview at the end of their MITI enrollment visit. After completing the MITI enrollment process, the enrolling MITI team member told patients about the study, assessed patient interest in completing an interview, and requested patient permission to page an onsite member of the study team. The study team member arrived to provide the patient with more information about the study and obtain participants’ written informed consent using an IRB-approved consent form.

#### Staff

We were interested in interviewing 20 staff members (10 per site). Our staff sampling frame was the staff at each site who had a role in MITI: physicians referring patients to MITI, nurses performing titration monitoring and giving instructions, and clinic administrators who were involved in implementing MITI. We used purposeful criterion sampling [14] to recruit the staff for interviews. Factors that informed our sampling approach included gender, staff role (provider—physician, nurse practitioner, or physician assistant; registered nurse; and administrator), site, and whether they had referred at least 1 patient to MITI (for providers) or assisted at least 1 patient through the MITI program (for nurses). The staff were invited to participate via institutional email and sign-up lists distributed during regular staff meetings. The staff had signed an IRB-approved consent form before interview procedures began.

### Interview Procedures

#### Conceptual Framework

Our interview and analytic approach was guided by the Consolidated Framework for Implementation Research (CFIR) [15]. The CFIR specifies 39 constructs mapping to 5 major domains that delineate the potential barriers and facilitators of implementation outcomes: (1) the *characteristics of an intervention*, (2) the *outer setting* of the organization in which the intervention is being implemented, (3) the *inner setting* of the organization, (4) the *characteristics of individuals* involved in the intervention, and (5) the *implementation process*. We conceptualized the *inner setting* for patients as being their personal setting (eg, home and work) where they would perform their MITI activities.

#### Patients

Patients were asked to complete 2 interviews—an interview immediately after the MITI enrollment and another 12 weeks later or when they were discharged from MITI (whichever occurred first). Patients had the option to complete the interviews in English or using a translator phone. Enrollment interviews were conducted in private, closed offices located in the primary care clinics at each site. Patients could complete the follow-up interview at the hospital or over the phone. The interviews were conducted by 3 study team members (ESR, SA, and DM) using an interview guide (Multimedia Appendix 1) informed by the CFIR that included structured questions with follow-up probes to assess patient perceptions of the MITI program, including how it compared with other options for insulin titration and factors that might enhance or impede their use of MITI. Follow-up interviews assessed patient experiences of actually using MITI, including challenges encountered and recommendations for improving the program. All interviews were audio-taped. When a translator phone was used during interviews, the translator phone was recorded. The patients received US $20 in cash for each completed interview.

#### Staff

The staff were asked to complete 2 interviews—an interview during the early implementation period (first 5 months) at their site and another approximately 6 months later. The staff interviews were conducted in their private offices by the same team members who conducted the patient interviews. The interviewers followed a guide (Multimedia Appendix 2) informed by the CFIR to assess the staff perceptions of MITI, including how it compares with usual care, how it worked within the regular clinic workflow, and factors that might enhance or impede the staff and patient use of MITI. Follow-up interviews further assessed staff experiences using MITI, challenges encountered, and recommendations for improving the program in the future. Interviews were audio-taped. The staff received a US $20 gift card for each interview completed.

### Data Analysis

Interview audio files were transcribed verbatim, removing identifiers at the time of transcription. For interviews conducted using the translator phone, the English portion of the interviews was transcribed (ie, the interview questions as asked by English-speaking interviewers and the participant responses as translated into English by the translators). Transcribers reviewed each audio file twice to confirm transcription accuracy. A total of two study team members (ESR and RF) used both deductive (CFIR theory–driven) and inductive (open coding) approaches to code the transcripts using Atlas.ti software (Scientific Software Development GmbH). An initial codebook was created that included all 39 CFIR constructs as codes. The codebook included a definition of each domain/code and its inclusion/exclusion criteria. The two coders independently coded 5 transcripts deductively using the CFIR codebook and with open coding to create preliminary codes specific to the study that were then mapped to the CFIR constructs. The coders met to discuss agreement and disagreement in the first round of coding and to collapse the preliminary codes to limit redundancy. Once they finalized the codebook (Multimedia Appendix 3), they completed independent coding of the remaining transcripts. Differences in coding were resolved via discussion. Once coding was complete, the coders met to identify themes (using memoing and reading of quotations to identify themes within codes and using frequency of code occurrence in the Atlas.ti dataset) and relationships among codes.

## Results

### Patient Participant Characteristics

Owing to delays in obtaining IRB approval for interviews at Gouverneur, our interview recruitment period was shorter than planned and we were only able to offer interview participation to 45 patients (39 of whom agreed to participate). The most common reason given for not participating was that the patient did not have time to stay for the interview. One patient did not want to be audio-taped and was not enrolled. The sociodemographics and program outcomes of the patient interview sample are shown in Table 1. The characteristics of the interview sample were similar to those in the broader MITI sample [10]. Participants at both sites were predominantly male, on average aged 49 to 51 years, and most reported Hispanic ethnicity. As was found in the general MITI population, participants in the interview sample were highly responsive to the program’s text and titration call procedures, and most graduated from MITI finding their OID with an FBG level within the goal of 80 to 130 mg/dL.

**Table 1:** Characteristics and Mobile Insulin Titration Intervention program outcomes of the patient interview sample.

Demographic characteristics			Bellevue (n=24)	Gouverneur (n=15)	Total (N=39)
Female gender, n (%)			8 (33)	6 (40)	14 (36)
Age (years), mean (SD)			51 (11)	49 (10)	50 (11)
**Race, n (%)**					
	White		4 (17)	2 (13)	6 (15)
	Black or African American		5 (21)	1 (7)	6 (15)
	Asian		2 (8)	1 (7)	3 (8)
	Native American/Alaskan Native		1 (4)	1 (7)	2 (5)
	Other^a^		12 (50)	10 (67)	22 (56)
Hispanic ethnicity, n (%)			16 (67)	13 (87)	29 (74)
Has health insurance, n (%)			10 (42)	12 (80)	22 (56)
Had a visit copay, n (%)			9 (38)	4 (27)	13 (33)
Unemployed, n (%)			12 (50)	4 (27)	16 (41)
Preferred Spanish language for texts, n (%)			11 (46)	8 (53)	19 (49)
**Mobile Insulin Titration Intervention program outcomes, %**					
	Text response rate		97	96	97
	Call connection rate		74	95	82
	**Program termination status, n (%)**				
		Achieved OID^b^	21 (88)	13 (87)	34 (87)
		Achieved 80-130 FBG^c^	18 (75)	12 (80)	30 (77)
		Reached maximum dose without reaching FBG goal	3 (13)	1 (7)	4 (10)
		Programmed terminated early	0 (0)	1 (7)	1 (3)
		Reached 12 weeks without OID	3 (13)	1 (7)	4 (10)

^a^All patients who checked the *Other* race option said they were Hispanic.

^b^OID: optimal insulin dose.

^c^FBG: fasting blood glucose.

In total, 25 patients (n=15 Bellevue and n=10 Gouverneur) completed a follow-up interview. The characteristics of follow-up respondents were similar to the full interview sample (mean age 51.5 SD [11.5] years; 36% female; 70% Hispanic; 60% with health insurance; 44% unemployed; 44% requested Spanish texts; and 80% achieved their OID).

### Staff Participant Characteristics

The study coordinator reached out to 32 staff to offer an interview. At Bellevue, 25 physicians, the 2 DNEs working on MITI, 2 physician assistants, 2 nurse practitioners, and 1 administrator were invited. At Gouverneur, 6 physicians, 17 nurses, and 1 administrator were invited. In total, 19 staff agreed to participate. Only 1 staff member actively refused participation; the other staff members could not be reached after multiple attempts. The final staff sample at Bellevue included 6 physicians (3 men and 3 women), 1 physician assistant (woman), and 1 clinic administrator (woman). The final staff sample at Gouverneur included 4 physicians (3 men and 1 woman), 6 nurses (5 women and 1 man), and 1 nursing administrator (woman). A total of 14 staff (n=7 per site) completed a follow-up interview.

### Facilitators

**Figure 1:**
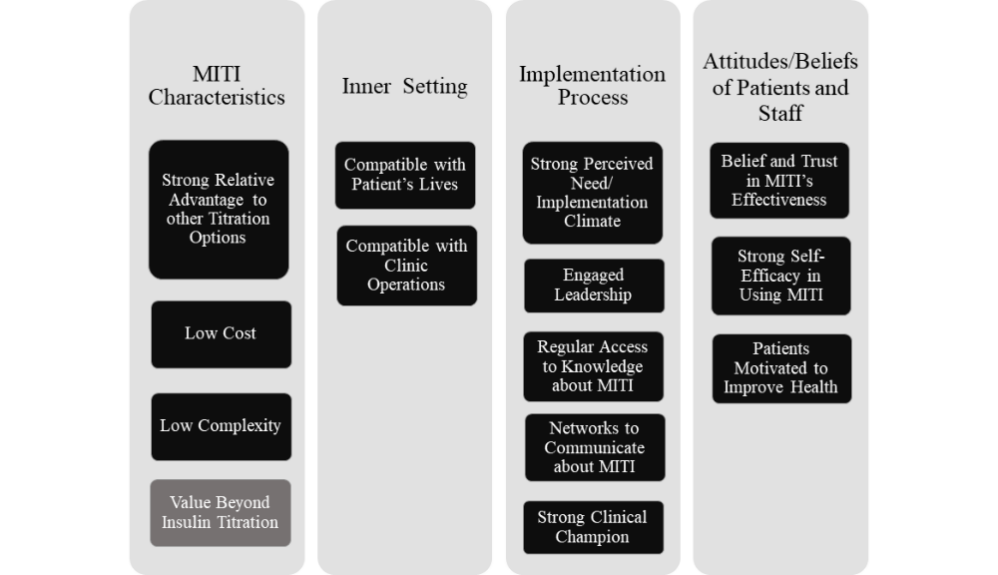
Themes related to factors that facilitated the implementation of Mobile Insulin Titration Intervention (MITI) at the two clinics.

Figure 1 displays the implementation facilitators organized around the main domains of the CFIR that emerged during the interviews. Facilitators in black boxes were deductively derived from the 39 CFIR constructs. Facilitators in gray boxes were derived from inductive open-coding and then mapped to the main domains of the CFIR. Multiple themes emerged identifying the *characteristics of MITI*, characteristics of the *inner setting*, *beliefs and attitudes of the patients and staff*, and characteristics of the *implementation process* that facilitated provider referrals, patient enrollments, and patient use of MITI. Of note, the same themes emerged during baseline and follow-up interviews; so, we have combined them in the Results section.

#### Facilitators: Mobile Insulin Titration Intervention Characteristics

##### Strong Relative Advantage to Other Titration Options

All patients spoke about the advantages of MITI compared with in-person insulin titration options, which contributed to their decision to enroll and stay in the MITI program. Advantages included MITI’s convenience and ability to save patients time by not having to go to the hospital. One patient described it as follows:

[MITI] makes my life much easier. I’m a security guard. I do 60-something hour weeks. I don’t have the time to burn a day off to come down here. So, [MITI] makes my life much easier.

B17, English-speaking patient at Bellevue

Additional advantages for patients relative to in-person titration included the ability of the MITI texting platform to keep a record of FBG levels so that patients do not have to keep track themselves and the included the perception that the frequent text-based contact with providers was a better way to communicate with their health care team. When asked about the option to create MITI as a secure website or smartphone app, most patients either preferred MITI to remain a texting program or were neutral. Some patients stated that they did not have access to (or had limited comfort with) the internet and/or phone apps, whereas some patients reported that texting was simply easier or faster than logging into an app or a Web-based program.

Similar to patients, all the staff saw advantages to the MITI program compared with in-person titration. These advantages drove their decision to refer the patients to MITI. Perceived advantages of MITI included its convenience for the patients. A physician assistant at Bellevue shared the following:

MITI is extremely convenient and great for patients because our patients unfortunately have a lot of barriers when it comes to seeking care…the fact that they can receive optimal care just by text messaging is very efficient, cost-effective, and a great idea for the patient.

B209

The staff reported additional advantages to in-person care including MITI’s ability to speed-up the titration process because patients do not need to wait months just to have their FBG logs reviewed:

With MITI patients get seen faster. Patients get the equivalent of three nursing visits in weeks as opposed to months.

B202, Bellevue physician

The staff further perceived that there were nonpatient advantages of MITI, including its ability to reduce the burden of titration visits on providers. A clinic administrator suggested that MITI had advantages to non-MITI patients and to the health care system more broadly by reducing the number of visits for insulin titration:

[With MITI] the waiting time for all patients here would reduce because there are less patients in the waiting room just for finger sticks. This improves satisfaction and retention.

B205, Bellevue

##### Low Cost

The free cost of MITI and the free daily testing strips were important to all patients interviewed. However, the patients had mixed opinions on whether they would have enrolled in MITI if there was a cost associated with the program. A total of 15 patients would not have enrolled, as one woman described:

I live by myself on a budget and even my medication, to be honest with you, some of the medication, I was calling the providers and companies to see if I can get coupons. So, I don’t think I would sign up [for MITI] if I had to pay.

G114, English-speaking patient at Gouverneur

On the contrary, 9 patients were unsure if they would enroll with a cost or said it would depend on the amount of cost. A total of 12 patients said they would have enrolled even if there were a charge, because MITI was ultimately beneficial for their health.

##### Low Complexity

All patients valued MITI’s simplicity and ease of use. Only 1 patient reported challenges using MITI but noted that it got easier over time. One patient said:

It was easy. It takes messaging, y’know, with these smart phones and the ability to make a noise and then you just get up and do your test and then put that information back in there.

B11, English-speaking patient at Bellevue

Similarly, all staff believed that MITI would be easy for patients to use, in particular because MITI requires little of the patient and uses the familiar, easy technology of texting. This perception of MITI’s ease for patients contributed to physician motivations for referring patients to the program.

##### Perceived Value Beyond Insulin Titration

Interviews also showed that some patients experienced MITI as having value beyond insulin titration. A total of 10 patients discussed how MITI made them more aware of their diabetes and reminded them to check their FBG levels and to take their medication. As one patient noted:

I’m going to do a lot better because now I’m going to be reminded to [check my sugar], and at the same time, it’s going to remind me to take my medication.

B34, Spanish-speaking patient at Bellevue

Most patients also expressed that even though MITI did not address healthy eating directly, the process of being reminded to check their sugar motivated them to eat well or engage in other healthy behaviors. One female patient noted that*:*

[MITI] reminded me about my sugar, so I have to control what I eat. Every time you text me, I remember okay, you can’t eat this, you can’t eat that.

G127, English-speaking patient at Gouverneur

Finally, 4 patients reported that the daily text messages and weekly phone calls from a nurse felt like a needed source of personal support. In the words of one patient:

I know the program will help me and I will get support, and that’s what I think I need: the support.

B39, English-speaking patient at Bellevue

Most staff also saw value in MITI beyond insulin titration, including MITI’s ability to engage with patients in between visits and potentially increase medication adherence by reminding the patients to attend to their diabetes and use their insulin daily. A physician also felt that by making the insulin titration process easy, MITI might reduce the burden on patients of starting insulin. In his words:

Starting insulin is always kind of like the biggest hurdle to overcome in a diabetic. I think having this program where they don’t have to come in to the clinic, it’s not a big intrusion, like a big change to their routine.

G301, Gouverneur physician

#### Facilitators: Inner Setting

##### Compatibility of Mobile Insulin Titration Intervention With Patients’ Lives

At enrollment, all patients felt that MITI would fit well with their regular routines at home and that they would need to make minimal, if any, changes to be able to send the daily text messages. The follow-up interviews confirmed that this expectation was accurate for most patients, who expressed that they had no challenges in sending the daily text. In addition, related to the *relative advantage* findings discussed above, MITI was more compatible with patients’ lives than in-person care. One patient noted that:

Part of the reason why I went missing for a year from the hospital is because I have to come down here…Whereas with MITI I can get a text message and then get a weekly phone call. I’m more likely to stay on top of it, because I can fit it into my schedule versus having to fit someone else’s schedule.

B17, English-speaking patient at Bellevue

All staff similarly viewed MITI as being compatible with patients’ lives, which contributed to their decision to refer patients. A physician said:

MITI is part of [patients’] routines because they’re already going to get up and check [their FBG] in the morning.

G302, Gouverneur

##### Compatibility of Mobile Insulin Titration Intervention With Clinic Operations

Most nurse interviews at Gouverneur (where nurses did not have protected time for MITI patients) revealed that managing the MITI patients worked well with the regular clinic work flow. Physician interviews at both sites also found that the process of referring the patients to MITI was easy:

MITI referral is almost entirely similar to what I do now, so it’s no problem.

G304, Gouverneur physician

#### Facilitators: Beliefs and Attitudes of Patients and Staff

##### Trust in Mobile Insulin Titration Intervention’s Effectiveness

Interviews revealed that most patients believed in the effectiveness of MITI at the time they enrolled. They were confident that MITI would help them improve their health and their diabetes management, which contributed to their decision to enroll. One patient told us:

There’s a good chance that I can probably get my diabetes under control and live a good healthy life. That’s what I’m looking forward to.

B11, English-speaking patient at Bellevue

Physicians also trusted and perceived the evidence supporting MITI’s potential effectiveness to be of high quality. Most interviewees were aware of the previous pilot RCT showing MITI to be efficacious compared with usual care. The staff also reported that the data and feedback that they received from the MITI program regarding the successes of patients whom they referred to MITI supported their decision to continue referring.

##### Strong Patient Self-Efficacy in Using Mobile Insulin Titration Intervention

There were a few patient concerns about their ability to use MITI (concerns are described in the *Barriers* section). All patients were highly confident in their ability to use MITI and send the daily text message. Patients reported that their high confidence was related to the simplicity of MITI and how well it fit with their routine:

It’s easy to text somebody…you know…that’s all you do all day… is text.

B24, English-speaking patient at Bellevue

##### Patients Motivated to Improve Health

Finally, some patients were highly motivated to improve their health. This motivation contributed to their decision to enroll in MITI and stay engaged in the program once enrolled. A woman told us:

I was very interested in [MITI] to get my blood sugar down, cause it’s been high for a long time…I’ll do anything that can help.

B24, English-speaking patient at Bellevue

A man similarly shared:

I’ve been a diabetic for better part of 20-25 years, when I really found out about it. Probably longer than that and I never paid attention to it. Now it’s getting down to that point where it needs to be a big concern.

B11, English-speaking patient at Bellevue

#### Facilitators: Implementation Process

##### Strong Perceived Need for Mobile Insulin Titration Intervention And Strong Implementation Climate

When it came to the process of implementing MITI, most staff reported that there was strong support among clinicians for the implementation of MITI. They recognized that this new service was meeting the needs of patients who could not attend frequent clinic appointments. A physician at Bellevue said:

There’s been overwhelming support for [MITI] because it really does facilitate the care of our patients. It’s a good alternative to regular clinic visits because it can be difficult to fit in regular clinic visits for something like insulin titration.

B207

##### Regular Staff Access to Knowledge About Mobile Insulin Titration Intervention

In addition, all staff interviewees were aware of MITI, and most reported that they had regular access to information about MITI. They reported receiving initial trainings and educational outreach for MITI. Most providers felt that the information provided to them about MITI was comprehensive and easy to understand. Gouverneur staff reported that there were information packets about MITI in the shared precepting and break rooms that were updated regularly. Nurses also appreciated the ongoing support provided by the MITI team when they had questions about the texting platform or other procedures. A nurse reported that:

[The MITI coordinator] would tell us that if anyone wants to practice enrollments or the texting program to come see her for a little bit. She would help whoever felt uncomfortable doing it on their own and practice with them.

G302, Gouverneur

##### Formal and Informal Networks to Communicate About Mobile Insulin Titration Intervention

All staff described that each clinic had opportunities for formal discussion about MITI, including regular staff meetings and emailed distribution of MITI information. The staff also reported that there was informal communication about MITI within the clinic, such as physicians hearing about MITI from colleagues or sharing patient successes. These formal and informal communication opportunities facilitated the sharing of information about MITI during and after implementation.

##### Strong Clinical Champion

Finally, 3 staff reported that the MITI coordinator and medical director (whom most interviewees knew by name) were strong champions for the program, which facilitated implementation. A Bellevue physician noted:

I’m impressed with everything accomplished, with overcoming bureaucratic hurdles with the texting and the patient protection of health information issues. It’s a real testament to the ability of [the MITI medical director]. It takes a champion.

B201

### Barrier Themes

**Figure 2:**
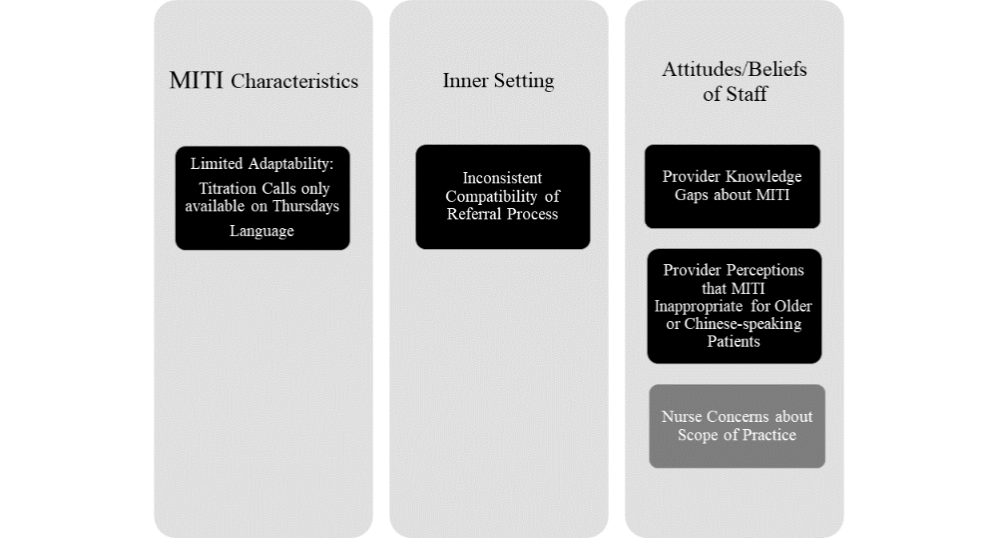
Themes related to barriers toward the implementation of Mobile Insulin Titration Intervention (MITI) at the two clinics.

Figure 2 displays the themes related to the implementation barriers organized around the main domains of the CFIR that emerged during interviews. Barriers in black boxes were deductively derived from the 39 CFIR constructs. Barriers in gray boxes were derived from inductive open-coding and then mapped to the main domains of the CFIR. A few barriers were discussed by the patients or staff. Those identified were related to the *characteristics of MITI*, *characteristics of the inner setting*, and *beliefs or attitudes of the staff*, which limited provider referrals or impeded patient use of MITI. These are described in detail below.

#### Barriers: Characteristics of Mobile Insulin Titration Intervention

##### Limited Adaptability

###### Titration Calls Only Available on Thursdays

Some patients expressed concern at being able to take the Thursday titration calls, because they had work commitments or other activities that would make it difficult to receive a call on Thursdays. They requested more flexibility in the ability to pick the day of week or time of day during which they would receive the titration calls.

###### Language Limitations

Multiple staff pointed out that MITI was not appropriate for their patients who could not text in English or Spanish, which could comprise a significant portion of patients at the program’s two safety net sites. A physician noted:

One aspect [of concern] was definitely language. I think MITI [only] included English and Spanish, so language can be a barrier for some.

G301, Gouverneur

#### Barriers: Inner Setting

##### Limits to Clinic Compatibility (Referral Process)

Once MITI launched at Bellevue, the process of paging the MITI coordinator was described as unusual by some staff because the clinical referral process typically happens through the electronic health record (EHR). Physicians requested the ability to refer via the EHR. At Gouverneur, interviewees noted that their team nurses were not always available to immediately enroll an MITI patient. Gouverneur nurse interviewees also suggested that physicians were often too busy to identify the patients who would be potentially eligible for MITI and offered to identify the potential MITI patients themselves.

#### Barrier: Beliefs or Attitudes of Staff

##### Initial Staff Knowledge Gaps About Mobile Insulin Titration Intervention

Interviews conducted in the early implementation period identified some staff knowledge gaps about MITI. Some staff were not aware of what MITI offered and did not offer (eg, insulin refills) or who to contact with questions about MITI.

Some staff also thought that MITI was still being tested as part of a clinic trial, were not aware that MITI was offered in Spanish, or were not aware that the Chinese-speaking patients could be referred to MITI as long as they were able to text in English or Spanish. Finally, nurses at Gouverneur reported that initially they were not sure how many times they should try to a reach a patient for weekly titration instructions. Of note, the MITI team responded to these knowledge gap findings by updating educational materials for the clinicians and clarifying MITI protocols. Follow-up interviews did not identify remaining knowledge gaps among the staff.

##### Nurse Concerns About Scope of Practice and Liability

In the early implementation period, all the interviewed nurses at Gouverneur were initially concerned about how their MITI responsibilities would fit with their regular workload and scope of practices, which were eventually modified to incorporate MITI. In addition, nurses were concerned about the need to frequently monitor and immediately respond to the patients or alarming FBG levels during busy clinic operations. A Gouverneur nursing administrator described these initial concerns and how the MITI team responded:

I think [the nurses’] biggest concern was if the patients contact the nurses and they can’t immediately respond back, or if there was a really grossly high or low blood sugar, that they would put the patient in danger by not being as attentive as they would want to be. What ultimately happened, which was really good, was the MITI lead explained that this kind of situation is [rare]. Once the nurses got those reassurances, and once they knew that I was here and the MITI team was consistent in showing up to provide support, it wasn’t that big of a lift.

G304

##### Physician Perceptions That Mobile Insulin Titration Intervention is Inappropriate for Some Patients

Some staff suggested that MITI is not appropriate for some of their patients, such as older patients or those who are not comfortable with texting. Some providers also perceived that their Chinese-speaking patients do not regularly text or have texting plans. Interviews revealed that these concerns about patient appropriateness of MITI impacted physician referral decisions. One Bellevue physician discussed his concerns:

Some of our patients also, even though they have a phone and they have text messaging available to them, they’re not high utilizers of that kind of technology, so sometimes I think the idea of using their smart phone to text is a bit new to them and also they may not be their primary mode of communicating so they may not be as comfortable with the idea of talking to someone about something like medication changes.

B201

### Additional Noteworthy Findings

There were 3 additional findings from the interviews that were not implementation barriers or facilitators but were noteworthy, nonetheless.

#### Patient and Staff Desire for Mobile Insulin Titration Intervention to Last Longer

Some patients and staff felt that patients needed a longer-term program than MITI. Patients and staff felt that being discharged from MITI after only one low FBG value was too soon, as patients might not be able to maintain the low value without ongoing support. In the words of one patient:

Let [MITI] go longer. Even though I got my numbers back in a short period of time, I still would like to see the program go longer just to make sure there’s no relapse. ’Cause I’m a diabetic, so of course I can relapse at any time.

G132, Spanish-speaking patient at Gouverneur

Similarly, some providers wanted MITI to last longer because the patients might have lifestyle factors or varying eating habits that caused fluctuating blood sugar:

It was a little unfortunate that as long as you just get one [low FBG value], then you’re out of the program. Sometimes people might have life factors, like they didn’t eat and then all of a sudden their sugar is low one time, but then the next day they’re back to their normal eating schedule and their sugar is 160. That might be an issue. We would usually tell people, even if [your sugar is] low one time, you keep the same dose and you keep on checking, and then if the next couple days it’s back to a higher level, you would still go up [in insulin] as opposed to just stopping where you were.

G301, Gouverneur physician

#### Unmet Need for Additional Diabetes Support

There was also a theme identified that MITI addressed just 1 component of diabetes care (insulin titration) and there was an unmet need for additional diabetes support more broadly in the population. In particular, 4 physicians noted the need for more services related to nutrition and exercise education and compliance. A Bellevue physician noted that:

In general, the diabetics who need insulin, they tend to be people who have very little control over when they eat…one of the big needs is lack of knowledge of diet and really a desire to know more about eating.

B205

#### Physician Desire to Expand Mobile Insulin Titration Intervention to Other Conditions

Finally, most providers discussed that they would like to see MITI expanded to other conditions, such as hypertension. One Bellevue physician noted that:

The broad idea of using mobile technology to adjust something like insulin is a new direction for care of chronic conditions. Seeing this apply to other things, like blood pressure, would be great in the future.

B105

## Discussion

### Principal Findings

This study was designed to understand the perspectives of patients and staff regarding potential barriers and facilitators to implementing the first mobile text message–based intervention for insulin titration (called MITI) into routine ambulatory care. The interviews revealed that the patients had highly favorable perceptions of the MITI program and positive experiences using the program, which contributed to their decision to enroll in and complete the program. Patients reported multiple advantages of MITI compared with in-person visits, especially its convenience. Patients also found the program easy to use and mostly compatible with their lives, which we believe is related to MITI’s simplicity (our *low complexity* finding). MITI is also a short program (most patients graduated in 2-3 weeks); thus, requiring no long-term engagement with the program and limiting the potential for message fatigue [16]. These findings are consistent with mHealth usability research suggesting that simplicity should be a primary feature of user-centered design [17]. However, these results also found that many patients and staff wanted MITI to last longer and expand to other chronic conditions. Therefore, future research should balance designing for simplicity and limiting message fatigue with the need to address the long-term nature of diabetes and other chronic diseases.

Diabetes-related distress is common in people with type 2 diabetes and can encompass many factors, such as the overwhelming emotional burden of having a serious medical condition, the burden of the diabetes regimen (eg, taking medications, checking FBG levels, and eating well), interpersonal distress, and stress associated with interacting with one’s health care providers [18]. This study found that patients valued MITI beyond insulin titration because MITI reminded and motivated them to achieve health goals and provided them with personal support. This was surprising, given that MITI involves a single daily text message asking for their FBG level and a weekly call focused solely on insulin instructions. Consistent with user-centered models supporting the power of simplicity, our results suggest that short, proactive communications with patients who have type 2 diabetes may be effective in alleviating diabetes-related distress and encouraging healthy behavior change, which can be assessed in future randomized trials.

Low adoption by providers can be one of the greatest barriers to implementation of mHealth interventions. This study found that the staff had favorable views about and experiences using MITI, which facilitated physician decisions to refer patients to the program and facilitated nurses’ work while executing the program. The staff results also suggest that physician adoption of MITI was determined primarily by their perceptions of MITI’s effectiveness and benefits to the patients—consistent with the research identifying factors that influence provider adoption of new telehealth interventions [19,20]. Future projects implementing MITI or other mHealth interventions may focus on communicating the interventions’ effectiveness and benefits to the patients to enhance provider adoption.

There was a notable strong concordance between most staff and patient perceptions of MITI. In particular, the patients and staff similarly believed MITI had advantages to in-person titration care, was compatible with the patient’s regular routines, was easy to use, and had value beyond insulin titration. The patients and staff also shared a desire for MITI to last longer. These shared perceptions might have synergistically facilitated MITI implementation and sustainability. In addition, future adaptations of MITI responding to this shared feedback would likely have high acceptability by both the patients and staff. Of note, there was also strong agreement in interview results at the two sites (which led us to combine site findings for this report), despite the two hospitals serving somewhat different patient populations and using a different MITI staffing model (ie, a MITI coordinator and DNEs with protected time at Bellevue; team nurses without protected time at Gouverneur). These results support the generalizability of MITI in different safety net settings and the ability of the two staffing models to function smoothly.

The interview findings differed between the patients and staff mostly when it came to discussing barriers. Although the patients and staff did not disagree about barriers, they experienced different barriers. On the patient side, some patients reported challenges taking the Thursday titration calls. As program flexibility can be a strong determinant of telehealth acceptability and adoption by patients [21], the MITI team is testing potential methods for improving patient-centeredness of the call process, including letting the patients pick the day and time of the week to receive a call if needed and sending titration instructions via text for low-risk patients. With regard to the staff, barriers reported by the staff were mostly encountered during early implementation, including staff knowledge gaps about MITI and nurse concerns about the new responsibilities and scope of practice. The MITI team was able to quickly address these initial challenges, and future sites should plan for them before or shortly after MITI implementation.

However, the staff interviews also identified 3 reasons providers might not have referred some patients to MITI: older age, non-English or Spanish language, and Chinese nationality. Although older adults are less likely to own a cell phone, ownership rates in people aged above 65 years are near 80%, and 90% of cell phone owners aged above 65 years use text messaging [22-24]. Therefore, age alone should not be a reason for not offering MITI to patients, and future MITI implementation work should address provider misperceptions about cell phone and text usage in older patients. We also heard from the staff that some patients were unable to understand English or Spanish texts, so the MITI team is exploring the translation of MITI’s text messages. Finally, a small number of the staff discussed that the Chinese-speaking immigrant patients might not use text messaging. However, the use of text messaging is widespread in China, and research supports the use of text messaging–based health interventions in China [25]. Nonetheless, reports suggest that the use of SMS text messaging has been declining in China, replaced by cheaper internet-based messaging services, such as WeChat [26]. In addition, in the United States, there is a growing popularity in smartphone apps and apps for diabetes care [27]. Future research should examine the acceptability, feasibility, and effectiveness of offering MITI using smartphone-based and Web-based messaging platforms. Of note, this study found that some patients would not have been able to use MITI if it were offered as a Web-based or smartphone program, because they lacked a smartphone or data plans. Therefore, to continue to serve very low-income populations, mHealth developers using smartphone technology should seek ways to increase patient access to smartphones and data plans or offer their programs in an SMS-based version when needed.

### Limitations

We were unable to interview the two diabetes nurses performing FBG monitoring and titration calls at Bellevue Hospital. We also did not interview patients with diabetes who were *not* enrolled in MITI. Therefore, results only reflect the perceptions of patients who were offered MITI by their physician, accepted the referral, and ultimately enrolled in the program. In addition, we were only able to conduct follow-up interviews with 25 of 39 patient participants, so there might be some bias in our findings related to the actual patient use of MITI. However, as described in the methods, the characteristics of follow-up respondents were similar to the general interview sample. Finally, some interviews were conducted using a translator phone, resulting in language discordance during the interview and the analysis process, which might have impacted the validity of qualitative results [25]. Additional language-concordant work should be conducted to further understand the perspectives of non–English-speaking patients.

### Conclusions

MITI is the first mHealth intervention designed to help patients with type 2 diabetes who need insulin adjustment. Our previous quantitative analysis showed MITI to be effective in helping patients find their OID, a lower FBG level, and a lower hemoglobin A_1c_ level in a short period of time [9]. These qualitative findings complement the quantitative results by showing that MITI patients and the staff were overwhelmingly supportive of MITI, believed it had many benefits, and encountered a few barriers to its use. Health care systems can use these results to design strategies for implementing MITI into their clinic workflows.

## Appendix

Multimedia Appendix 1 Patient interview guide.

Multimedia Appendix 2 Staff interview guide.

Multimedia Appendix 3 Codebook.

## Abbreviations

CFIR: Consolidated Framework for Implementation Research
DNE: diabetes nurse educator
EHR: electronic health record
FBG: fasting blood glucose
IRB: institutional review board
mHealth: mobile health
MITI: Mobile Insulin Titration Intervention
NYC: New York City
OID: optimal insulin dose
RCT: randomized controlled trial
SMS: short message service
