# Supplementary material for: Barriers and Facilitators to the Implementation of a Mobile Insulin Titration Intervention for Patients With Uncontrolled Diabetes: A Qualitative Analysis
Source: JMIR Mhealth Uhealth. 2019 Jul 31;7(7):e13906. doi: 10.2196/13906 (PMC6693299; doi:10.2196/13906)
Supplement: Multimedia Appendix 1 [file mhealth_v7i7e13906_app1.docx]

**MITI**

**Patient Interview Guides**

Study ID: ______________________

Interview Date:

Interview Language: _____________

Interviewer initials:

**Baseline Patient Interview Guide**

**I. Welcome**

Thank you for taking part in this interview. My name is _______ and I am a researcher at the NYU School of Medicine. The purpose of this session is to hear your views and opinions about a text-messaging program called MITI that the hospital is offering to patients with diabetes. Your insights are very important to us and your time today is appreciated.

Before we begin, I want to let you know that there are no right or wrong answers. We want to know your opinions and what you think. If at any time you are uncomfortable with my questions, you can choose not to answer. Simply let me know that you prefer not to answer.

Do you have any questions before we begin?

*{Start Recording}*

**II. Introduction**

You were recently enrolled in the MITI text-messaging program that will ask you obtain the best insulin dose for your diabetes. As I mentioned before, we are conducting a study to learn what patients like about the program, what they may not like, and what we can do to make the program better. Before I begin asking your questions about MITI, I’d like to ask a few pieces of information about you.

**III. Patient Demographics**

1. What is your age? ________
2. What gender do you consider yourself?

- Male
- Female
- Transgender Male to Female
- Transgender Female to Male
- Other

1. What is your race? You can choose more than one.

- White
- Black or African American
- American Indian or Alaska Native
- Asian
- Native Hawaiian/Pacific Islander
- Some other race (specify): __________________

1. Are you of Hispanic, Latino, or Spanish origin?

- No (not of Hispanic, Latino, or Spanish origin)
- Yes (of Hispanic, Latino or Spanish origin)

1. What type of health insurance do you have (check all that apply)?

- Medicaid
- Medicare
- Private Insurance
- Other public insurance (i.e. Tricare, Champus)
- None

**IV. FACILITATORS AND BARRIERS TO USING MITI**

Thank you for that information. Now I’d like to begin the questions about MITI.

**CFIR domains: MITI characteristics, Patient Beliefs/Attitudes, Outer Setting, Process**

1. **When your health care provider recommended the MITI program to you, what was your first impression of the program?** PROBES: How did you feel about the information or materials given to you about the program? How did you feel about the training given to you to sign-up for MITI? Is there anything else you would have liked to know about the program?
2. **What are some reasons you decided to enroll in the MITI program?** PROBES: What did you like most about the program? Did you have any concerns about signing up? Is there anything you could recommend to make the enrollment process easier for patients? How did you feel about being able to communicate with your health care provider via text? How did you feel about the fact that the texting program was free? Would you have enrolled if you had to pay for the texts?
3. **How complicated or uncomplicated do you think it will be to use the program?** PROBES: What are some reasons you think it will be easy? What are some reasons you think it will be hard?
4. **How well do you think the MITI program will work in terms of helping you and your doctor find the right amount of insulin for you?** PROBES: what makes you feel that way?
5. **When you signed up for MITI, you were told that text messages are not totally private. How do you feel about the privacy of the text messages? PROBES:** How would you feel if someone saw the MITI text messages on your phone?
6. **How does MITI compare to other options you are aware of for helping you find the best insulin dose?** PROBES: What advantages does MITI have compared to other options? What disadvantages does MITI have?
7. **The MITI program may also become available to use through a website or smartphone ‘app’ instead of texting. How would you feel about using a website or phone app to talk to your doctor or nurse instead of texting?** PROBES: What makes you interested/not interested? What would make a website or app easier/not was easy for you?

**CFIR Domains: Inner Setting**

1. **As your doctor or nurse discussed with you, the MITI program will ask you to send a text message each morning after you take your morning sugar level. How confident or not confident are you that you will be able to send the message every morning?** PROBES depending on answer: What are some reasons you are confident? What are some of your concerns about being able to send a daily text? What would make you more confident in being able to send the text every day? What kinds of changes, if any, to your nightly or morning routine do you think you will need to make to send the texts?
2. **Part of the MITI program will include the MITI nurse calling you every Thursday during hospital hours. How do you feel about being able to take those Thursday calls?** PROBES: Do you have any scheduling conflicts that would prevent you from answering your phone on Thursdays? What kinds of changes, if any, to your routine do you think you will need to make to speak with a nurse on Thursdays?

**OPEN**

1. **Is there anything we haven’t talked about that you think would be helpful for us to know as we try to improve the MITI program for other patients?**

**VI. Closing**

We’re all done with the discussion now. Thank you very much for participating today. We will be calling you in a few weeks to ask you to do a second interview. If your phone number changes, please call our office to let us know.

***{Stop recording}***

**Audio file name:**

Study ID: ______________________

Interview Date:

Interview Language: _____________

Interviewer initials:

**Follow-up Patient Interview Guide**

**I. Welcome**

Thank you for taking part in this interview. My name is _______ and I am a researcher at the NYU School of Medicine. The purpose of this session is to hear your views and opinions about a text-messaging program called MITI that you used. Your insights are very important to us and your time today is appreciated.

Before we begin, I want to let you know that there are no right or wrong answers. We want to know your opinions and what you think. If at any time you are uncomfortable with my questions, you can choose not to answer. Simply let me know that you prefer not to answer.

Do you have any questions before we begin?

***{Start recording}***

**II. Introduction**

You used the MITI text-messaging program to help you obtain the best insulin dose for your diabetes. As I mentioned before, we are conducting a study to learn what patients like about the program, what they may not like, and what we can do to make the program better.

**III. barriers AND FACILIATORS toward using the MITI intervention**

**CFIR domains: MITI characteristics, Patient Beliefs/Attitudes, Outer Setting**

1. **What was your experience using the MITI program?** PROBES: What did you like most about it? Did you experience any challenges in using MITI? How easy or hard was MITI to use?
2. **How well or not well do you think the MITI program met your needs in finding your best insulin dose?** PROBES: In what ways did the program meet your needs? How did MITI compare to other options for finding the best insulin dose (advantages, disadvantages)?
3. **When you signed up for MITI, you were told that text messages are not totally private. While using MITI, how did you feel about sending health information via text?** PROBES: What were your concerns? (or why did you not feel concerned?)

**CFIR domains: Inner Setting**

1. **Some patients miss sending the text message of their blood sugar level every now and then. Did you have any challenges in sending the text message every day?** PROBES: What are some reasons you were *not* able to send a text every morning? Or, what are some reasons you *were* able to send the text every morning? Did you have to make any changes to your daily routine to be able to send the daily text message?
2. **Some patients miss the Thursday calls with the nurse. Did you have any challenges being able to take the Thursday phone calls with the nurse?** PROBES: *{If patient reported challenges}*: Tell me more about that… What are some reasons you were not able to take the calls? What changes would you suggest be made to MITI to help patients take those Thursday calls?

**OPEN**

1. **Would you recommend MITI for other patients?** PROBES: What are some reasons for your recommendation (or for not wanting to recommend)?
2. **Is there anything we haven’t talked about that you think would be helpful for us to know about your experience using MITI?**

**IV. Closing**

We’re all done with the discussion now. Thank you very much for participating today and in the previous interview.

***{Stop Recording}***

**Audio file name:**
