# Supplementary material for: Barriers and Facilitators to the Implementation of a Mobile Insulin Titration Intervention for Patients With Uncontrolled Diabetes: A Qualitative Analysis
Source: JMIR Mhealth Uhealth. 2019 Jul 31;7(7):e13906. doi: 10.2196/13906 (PMC6693299; doi:10.2196/13906)
Supplement: Multimedia Appendix 2 [file mhealth_v7i7e13906_app2.doc]

**MITI**

**Staff Interview Guides**

Study ID: ______________________

Interview Date: __________________

Interviewer Initials: _______________

**Early Implementation Staff Interview Guide**

**I. Welcome**

Thank you for taking part in this interview. My name is _______ and I am a researcher at the NYU School of Medicine. The purpose of this session is to hear your views and opinions about a text-messaging program called MITI that the hospital is offering to patients with diabetes. Your insights are very important to us and your time today is appreciated.

Before we begin, I want to let you know that there are no right or wrong answers. We want to know your opinions and what you think. If at any time you are uncomfortable with my questions, you can choose not to answer. Simply let me know that you prefer not to answer.

Do you have any questions before we begin?

***{Starting Recording}***

**II. Staff Characteristics**

Before I begin asking your questions about MITI, I’d like to ask a couple pieces of information about you. This allows us to report in the aggregate who we interviewed, such as 5 nurses, 3 physicians… Is this OK with you?

1. What service or department do you work in?
2. What is your clinical or professional specialty?

**III. MITI Characteristics; staff Knowledge, Perceptions, Beliefs about MITI**

Great, thank you. I will now start my questions about the MITI program.

1. **Have you heard about the MITI program? IF yes, what have you been told? PROBES:** Where did you first learn about MITI? Who provided you with this information?
2. **What were your first impressions when you learned about MITI?**
3. **How does MITI compare to existing options for insulin titration? PROBES:** What advantages does MITI have compared to existing programs? What disadvantages does MITI have compared to existing programs?
4. **Is there another program that you or your coworkers would rather implement to help patients titration insulin?** PROBES: Can you describe that program? What are some reasons you or others would prefer to implement that program?
5. **OUTER SETTING: How well or not well do you think MITI will meet the needs of diabetes patients starting insulin? PROBES:** In what ways will the intervention meet their needs? In what ways will it not meet their needs?
6. **How easy or complicated do you think MITI will be for patients to use?** PROBES: What challenges will patients face to using MITI? What will be the easiest parts for patients using MITI? Are you aware of resources that can help patients having difficulty with using MITI?

**IV. IMPLEMENTATION PROCESS**

1. **What training meetings or materials have you been provided about MITI?** PROBES: How did you feel about the trainings or materials given to you about MITI? What else would you have liked to know about it when you were first trained or informed of MITI?

**V. INNER SETTING**

1. **What level of endorsement or support, if any, of MITI have you seen or heard from leadership in your clinic?** PROBES: What about from your colleagues? Does the level f support from leadership or colleagues impact your perception of MITI? If yes, how so?
2. **What are your roles in providing MITI to patients?**
3. **How do you feel about being able to successfully perform your role with MITI?** PROBES: How prepared or unprepared do you feel? How complicated or easy do you feel it will be to perform these new responsibilities?
4. **How well or not well do you think MITI fits with existing work processes and practices in your setting?** PROBES: Can you describe how MITI was integrated into your current workflow? Can you think of any issues or complications that may arise when making MITI referrals? What kinds of changes or alterations do you think you will need to make to make MITI work effectively in your setting?
5. ***{Only ask if staff is involved in patient referrals}:* How do patients respond to being referred to MITI?** PROBES: What have their concerns been? What have they liked about it? Can you give an example of a patient you referred to MITI?

**OPEN**

1. **Is there anything we haven’t talked about that you think would be helpful for us to know about MITI?**

**Extra Questions for Leadership Stakeholders**

1. **Did any local, state, or national performance measures, policies, regulations, or guidelines influence the decision to implement MITI?** PROBES: How will the MITI affect your organization's ability to meet these measures, policies, regulations, or guidelines?
2. **What kinds of infrastructure changes were needed to accommodate MITI?** PROBES:Provider scope of practice? Changes in formal policies? Changes in information systems or electronic records systems? Other?

***{Stop recording}***

**Audio File name:**

Study ID: ______________________

Interview Date:

Interivewer Initials:

**Follow-up Staff Interview Guide**

**I. Welcome**

Thank you for taking part in this interview. My name is _______ and I am a researcher at the NYU School of Medicine. The purpose of this session is to hear your views and opinions about a text-messaging program called MITI that the hospital starting offering to patients with diabetes a few months ago. Your insights are very important to us and your time today is appreciated.

Before we begin, I want to let you know that there are no right or wrong answers. We want to know your opinions and what you think. If at any time you are uncomfortable with my questions, you can choose not to answer. Simply let me know that you prefer not to answer.

Do you have any questions before we begin?

*{Start Recording}*

**II. Staff Characteristics**

Before I begin asking your questions about MITI, I’d like to ask a couple pieces of information about you. This allows us to report in the aggregate who we interviewed, such as 5 nurses, 3 physicians… Is this OK with you?

1. What service or department do you work in?
2. What is your clinical or professional specialty?

**III. MITI CHARACERISTICS; Knowledge, Perceptions, Beliefs about MITI**

Great, thank you. I will now start my questions about the MITI program.

1. **I’m going to start by asking a really broad question. What has your experience been so far with the MITI program for patients?** PROBES: About how many patients have you talked to/worked with so far with MITI? Can you provide some examples? What kind of feedback, if any, from patients have you heard about MITI? What kind of feedback, if any, from coworkers have you heard about MITI?
2. **How has MITI compared to existing programs in your setting for helping patients with insulin titration?** PROBES: What advantages has MITI had compared to existing programs? What disadvantages has MITI had compared to existing programs? Can you provide some examples?
3. **How well or not well do you think MITI has been in meeting the needs of diabetes patients?** PROBES: In what ways did MITI meet their needs? What changes would you recommend to improve the ways in which MITI meets the needs of patients?
4. **How have patients responded to being offered MITI?** PROBES: What concerns did they have? What did they like most about MITI? Can you give an example?
5. **How easy or complicated do you think MITI has been for patients?** PROBES: What challenges have you heard from patients about using MITI? What were some of the easiest parts for patients in using MITI?

**IV. INNER SETTING**

1. **How well or not well has MITI fit with existing work processes and practices in your setting?** PROBES: What issues or complications have arisen? How easy or difficult was it to incorporate new MITI procedures into your weekly work flow? In what ways?
2. **What has your role been in providing MITI to patients?** PROBES: Did you feel you had the resources to take on this new role? In what ways? How prepared do you feel you were to use/offer MITI? In what ways?
3. **What changes, if any, did you need to make to make MITI work effectively in your setting**? PROBES: Did you feel you had the ability to make these changes? Who did you go to when you needed to make changes

**OPEN**

1. **Is there anything we haven’t talked about that you think would be helpful for us to know about MITI?**

***Stop Recording}***

**Audio File**
