# Supplementary material for: Barriers and Facilitators to the Implementation of a Mobile Insulin Titration Intervention for Patients With Uncontrolled Diabetes: A Qualitative Analysis
Source: JMIR Mhealth Uhealth. 2019 Jul 31;7(7):e13906. doi: 10.2196/13906 (PMC6693299; doi:10.2196/13906)
Supplement: Multimedia Appendix 3 [file mhealth_v7i7e13906_app3.docx]

**MITI Qualitative Codebook – CFIR Codes**

| **MITI CHARACTERISTICS** |  |
| --- | --- |
| **Code** | **Definition** |
| 1. **Innovation Source** | Definition: Perception of key stakeholders about **whether MITI was externally or internally developed.**  Inclusion Criteria: Include statements about the source of MITI and the extent to which interviewees view MITI as internal to the organization.  Exclusion Criteria: Exclude statements related to **who participated in the decision process to implement MITI**. |
| 1. **Evidence Strength & Quality** | Definition: Stakeholders’ perceptions of the **quality and validity of evidence supporting the belief that MITI will have desired outcomes.**  Inclusion Criteria: Include statements regarding **awareness of evidence and the strength and quality of evidence**, as well as the **absence of evidence or a desire for different types of evidence**, such as pilot results instead of evidence from the literature.  Exclusion Criteria: Exclude or double code statements regarding the receipt of evidence as an engagement strategy. Exclude or double code descriptions of use of results from local or regional pilots to Trialability. |
| 1. **Relative Advantage** | Definition: Stakeholders’ perception of the **advantage of MITI versus an alternative solution**.  Inclusion Criteria: Include statements that demonstrate MITI is better (or worse) than existing programs.  Exclusion Criteria: Exclude statements that demonstrate a strong need for MITI and/or that the current situation is untenable and code to Tension for Change. |
| 1. **Adaptability** | Definition: The **degree to which MITI can be adapted, tailored, refined, or reinvented to meet local needs**.  Inclusion Criteria: Include statements regarding the (in)ability to adapt MITI to their context, e.g., complaints about the rigidity of the protocol.  Exclusion Criteria: Exclude or double code statements that **MITI did or did not need to be adapted** to Compatibility. |
| 1. **Trialability** | Definition: The **ability to test MITI on a small scale** in the organization, and to be able to reverse course (undo implementation) if warranted.  Inclusion Criteria: Include statements related to whether the site piloted MITI in the past or has plans to in the future, and comments about whether they believe it is (im)possible to conduct a pilot.  Exclusion Criteria: Exclude **descriptions of use of results** from pilots to Evidence Strength & Quality. |
| 1. **Complexity** | Definition: **Perceived difficulty of MITI**, reflected by duration, scope, radicalness, disruptiveness, centrality, and intricacy and number of steps required to implement.  Inclusion Criteria: Code statements regarding the **complexity of MITI itself**.  Exclusion Criteria: Exclude statements regarding the **complexity of implementation**. |
| 1. **Design Quality & Packaging** | Definition: Perceived excellence in how **MITI is bundled, presented, and assembled**.  Inclusion Criteria: Include statements regarding the **quality of the materials and packaging**.  Exclusion Criteria: Exclude statements about presence/absence of materials (code to Available Resources). Exclude statements about receipt of materials as an stakeholder engagement strategy. |
| 1. **Cost** | Definition: **Costs of MITI and costs associated with implementing** MITI including investment, supply, and opportunity costs.  Inclusion Criteria: Include statements related to the cost of MITI and its implementation.  Exclusion Criteria: Exclude statements related to physical space and time, and code to Available Resources. exclude statements related to costs of conducting the research components. |
| **Outer Setting** |  |
| 1. **Needs & Resources of Those Served by the Organization** | Definition: The extent to which the **needs of those served by the organization (e.g., patients)**, as well as barriers and facilitators to meet those needs, are **accurately known and prioritized by the organization.**  Inclusion Criteria: Include statements demonstrating **(lack of) awareness of the needs and resources of those served by the organization**.  Exclusion Criteria: Exclude statements that demonstrate a strong need for MITI and/or that the current situation is untenable and code to Tension for Change.  Exclude statements related to engagement strategies and outcomes, e.g., how innovation participants became engaged with MITI. |
| 1. **Cosmopolitanism** | Definition: The degree to which an organization is **networked with other external organizations.**  Inclusion Criteria: Include descriptions of outside group memberships and networking done outside the organization.  Exclusion Criteria: Exclude statements about general networking, communication, and relationships in the organization, such as descriptions of meetings, email groups, or other methods of keeping people connected and informed, and statements related to team formation, quality, and functioning, and code to Networks & Communications. |
| 1. **Peer Pressure** | Definition: **Mimetic or competitive pressure to implement MITI**, typically because most or other key peer or competing organizations have already implemented or are in a bid for a competitive edge.  Inclusion Criteria: Include statements about perceived pressure or motivation from other entities or organizations in the local geographic area or system to implement MITI. |
| 1. **External Policy & Incentives** | Definition: A broad construct that includes **external strategies to spread innovations** including policy and regulations (governmental or other central entity), external mandates, recommendations and guidelines, pay-for-performance, collaboratives, and public or benchmark reporting.  Inclusion Criteria: Include descriptions of external policies, performance measures and incentives. |
| **Inner Setting** |  |
| 1. **Structural Characteristics** | Definition: The social architecture, age, maturity, and size of an organization.  Inclusion Criteria:Include descripitions of the **structure of the organization**. |
| 1. **Networks & Communications** | Definition: The nature and quality of webs of social networks, and the nature and quality of formal and informal **communications within an organization**.  Inclusion Criteria: Include statements about **general** networking, communication, and relationships in the organization, such as descriptions of meetings, email groups, or other methods of keeping people connected and informed, and statements related to team formation, quality, and functioning.  Exclusion Criteria: Exclude statements related to implementation leaders' and users' access to knowledge and information regarding using **MITI** - i.e., training on the mechanics of MITI and code to Access to Knowledge & Information. Exclude statements related to engagement strategies and outcomes, e.g., how key stakeholders became engaged with MITI and what their role is in implementation. |
| 1. **Culture** | Definition: Norms, values, and basic assumptions of a given organization.  Inclusion Criteria: Include statements related to the **organizational culture of the setting** in which MITI was implemented.  Exclusion Criteria: Exclude statements related to patient or staff **personal culture** outside of the workplace. |
| 1. **Implementation Climate** | Definition: The capacity for change, shared receptivity of involved individuals to MITI, and the extent to which use of that innovation will be rewarded, supported, and expected within their organization.  Inclusion Criteria: Include statements regarding the **general level of receptivity to implementing MITI**. |
| 1. **Tension for Change** | Definition: The degree to which stakeholders **perceive the current situation as intolerable or needing change**.  Inclusion Criteria: Include statements that **(do not) demonstrate a strong need for MITI and/or that the current situation is untenable**, e.g., statements that MITI is absolutely necessary or that MITI is redundant with other programs. Note: If a participant states that MITI is redundant with a preferred existing program, (double) code lack of Relative Advantage, see exclusion criteria below.  Exclusion Criteria: Exclude statements regarding specific needs of individuals that demonstrate a need for MITI, but do not necessarily represent a strong need or an untenable status quo, and code to Needs and Resources of Those Served by the Organization. Exclude statements that demonstrate MITI is better (or worse) than existing programs and code to Relative Advantage. |
| 1. **Compatibility** | Definition: The degree of **fit** between the meaning and values attached to MITI by involved individuals, how those align with individuals’ own norms, values, and perceived risks and needs, and **how MITI fits with existing workflows, systems and patients’ lives or routines.**  Inclusion Criteria: Include statements that demonstrate the **level of compatibility MITI** has with organizational values and work processes. Include statements that demonstrate level of compatibility that MITI has with patients’ lives. Include statements that MITI did or did not need to be adapted as evidence of compatibility or lack of compatibility.  Exclusion Criteria: Exclude or double code statements regarding **the priority** of MITI based on compatibility with organizational values to Relative Priority. |
| 1. **Relative Priority** | Definition: Individuals’ shared perception of the **importance of MITI implementation** within the organization.  Inclusion Criteria: Include statements that reflect the r**elative priority of MITI**, e.g., change fatigue in the organization due to implementation of many other programs.  Exclusion Criteria: Exclude or double code statements regarding the priority of MITI based on compatibility with organizational values to Compatibility, e.g., if MITI is not prioritized because it is not compatible with organizational values. |
| 1. **Organizational Incentives & Rewards** | Definition: **Extrinsic incentives** to implement MITI such as goal-sharing, awards, performance reviews, promotions, and raises in salary, and less tangible incentives such as increased stature or respect.  Inclusion Criteria: Include statements related to whether organizational incentive systems are in place to foster (or hinder) implementation, e.g., rewards or disincentives for staff engaging in MITI. |
| 1. **Goals & Feedback** | Definition: The degree to which **the organizations’** **MITI goals are clearly communicated, acted upon, and fed back to staff**.  Inclusion Criteria: Include statements related to the (lack of) alignment of implementation and MITI goals with larger organizational goals, as well as feedback to staff regarding those goals, e.g., regular audit and feedback showing any gaps between the current organizational status and the goal.    Exclusion Criteria: **Exclude statements that refer to the implementation team’s (lack of) assessment** of the progress toward and impact of implementation, as well as the interpretation of outcomes related to implementation. |
| 1. **Learning Climate** | Definition: A climate in which: 1. Leaders express their own fallibility and need for team members’ assistance and input; 2. Team members feel that they are essential, valued, and knowledgeable partners in the change process; 3. Individuals feel psychologically safe to try new methods; and 4. There is sufficient time and space for reflective thinking and evaluation.  Inclusion Criteria: Include statements that support (or refute) the degree to **which the organization exhibits a “learning climate” in general (not specific to MITI).** |
| 1. **Readiness for Implementation** | Definition: Tangible and immediate indicators of **organizational commitment to its decision to implement MITI.**  Inclusion Criteria: Include statements regarding the **general level of readiness for MITI implementation**. |
| 1. **Leadership Engagement** | Definition: Commitment, involvement, and accountability of leaders and managers with the implementation of MITI.  Inclusion Criteria: Include statements regarding the **level of engagement of organizational leadership.** |
| 1. **Available Resources** | Definition: The **level of resources organizational dedicated for MITI implementation** and on-going operations including physical space and time.  Inclusion Criteria: Include statements related to the presence or absence of **resources specific to MITI** that is being implemented.  Exclusion Criteria: Exclude statements related to training and education and code to Access to Knowledge & Information. Exclude statements related to the quality of materials and code to Design Quality & Packaging. In a research study, exclude statements related to resources needed for conducting the research components (e.g., IRB applications, consenting patients). |
| 1. **Access to Knowledge & Information** | Definition: Ease of access to digestible information and knowledge about MITI and how to incorporate it into work tasks.  Inclusion Criteria: Include statements related to implementation leaders' and users' access to knowledge and information regarding use of the program, i.e., training on the mechanics of the program.  Exclusion Criteria: Exclude statements about general networking, communication, and relationships in the organization (e.g., meetings, email groups, or other methods of keeping people connected and informed), and statements related to team formation, quality, and functioning, and code to Networks & Communications. |
| 1. **Beliefs and attitudes about FMITI** | Definition: **Individuals’ attitudes toward and value placed on MITI**.  Inclusion Criteria: Individuals’ attitudes toward and value placed on MITI.  Exclusion Criteria: Exclude statements related to **familiarity with evidence** about MITI and code to Evidence Strength & Quality. |
| 1. **Self-efficacy** | Definition: Individual **belief in their own capabilities** to execute courses of action to achieve implementation goals. |
| 1. **Individual Stage of Change** | Definition: Characterization of the **phase an individual is in**, as s/he progresses toward skilled, enthusiastic, and sustained use of MITI. |
| 1. **Individual Identification with Organization** | Definition: A broad construct related to **how individuals perceive the organization**, and their relationship and degree of commitment with that organization. |
| 1. **Other Personal Attributes** | Definition: A broad construct to include **other personal traits** such as tolerance of ambiguity, intellectual ability, motivation, values, competence, capacity, and learning style. |

**MITI Qualitative Codebook – Open Coding**

| **Code** | **Subcodes** | **Definition** |
| --- | --- | --- |
| **Positive attitudes about MITI** | n/a | This is a broad theme capturing any statements that indicate the interviewee holds positive attitudes about MITI. |
| **Negative attitudes about MITI** | n/a | This is a broad theme capturing any statements that indicate the interviewee holds negative attitudes about MITI. |
| **MITI is more than insulin titration** | See below | Statements that indicate the interviewee perceives or believes that MITI has components or benefits beyond helping a patient and his/her doctor titrate insulin.  Exclusion Criteria: Exclude statements that demonstrate a need or desire for services beyond insulin titration and code as “unmet need for diabetes care.” |
|  | **MITI helps patient stay conscious of diabetes or health** | Statements that indicate the interviewee perceives or believes that MITI helps them stay conscious of diabetes, health, self-management |
|  | **MITI reminds patient to eat well or engage in other healthy behaviors** | Statements that indicate the interviewee perceives or believes that MITI reminds patients to eat well or engage in other health promoting-behaviors |
|  | **MITI helps with medication management/adherence** | Statements that indicate interviewee beliefs MITI will help with medication management and patient’s medication adherence. |
| **Provider/staff/patient knowledge gaps about MITI** |  | Statements that indicate the interviewee lacks knowledge about MITI. This could include lack of awareness of MITI at all, as well as about specific aspects of MITI such as eligibility criteria, staff, operations or services provided.  Exclusion Criteria: Exclude statements that demonstrate general, not MITI-specific, knowledge gaps. |
| **MITI represents the future of chronic disease management** |  | Statements that indicate the stakeholder believes MITI aligns with innovations in chronic disease management (e.g., mobile/telemedicine, population-based care, patient-centered care, value-based payment models).  Exclusion Criteria: Exclude statements about innovations chronic disease management in general that do not specifically tie MITI to those innovations. |
| **Beliefs about whether MITI will be clinically effective** | See below | Statements that speak to interviewee’s beliefs about whether MITI will be effective at improving patient health, achieving optimal insulin dose. Subcode belief valency (positive, negative ambivlanet). |
|  | **Belief that MITI will be effective** | Statements that indicate interviewee believes that MITI will be effective at improving patient health, achieving optimal insulin dose. |
|  | **Belief that MITI will not be effective** | Statements that indicate interviewee does not believe that MITI will be effective at improving patient health, achieving optimal insulin dose. |
|  | **Ambivalence about whether MITI will be effective** | Statements that indicate interviewee is unsure about whether MITI will be effective at improving patient health, achieving optimal insulin dose. |
| **Patient schedule** | See below | This is a broad category that captures statements that discuss patient’s schedule as it relates to MITI. Subcode specifics of the discussion. |
|  | **Belief that MITI will fit well with patient’s schedule/daily routine.** | Statements of belief that MITI will fit with patient’s schedule or daily routine. |
|  | **Concerns about how MITI will fit with schedule/daily routine.** | Statements of concern about whether MITI will fit with patient’s schedule or daily routine. |
| **MITI education/training** | See below | This is a broad theme capturing any statements about the interiewee’s beliefs about the education, training or informational materials they received about MITI. Subcode specific beliefs. |
|  | **Belief that MITI education was good/easy to understand** | Statements indicating that the interviewe believes that the MITI education, training or informational materials they receive was good or easy to understand |
|  | **Belief that MITI education was not good/easy to understand** | Statements indicating that the interviewe believes that the MITI education, training or informational materials they receive was not good or easy to understand |
| **Diabetes management is expensive/burdensome** | See below | Statements indicating that the interviewe believes that diabetes management is expensive and/or burdensome for patients. Expense could include real costs (e.g., test strips) or opportunity costs (e.g., missed word to go to appointments). |
|  | **Test strips are expensive, barrier to daily testing** | Statements about the high expese of testing strips. Include statements that indicate patient believes the cost of testing strips is a barrier to daily testing. |
|  | **Patient would have enrolled in MITI if it were not free.** | Statement that patient would have enrolled in MITI even if there was a cost. |
|  | **Free cost of MITI is important/Patient would NOT have enrolled in MITI if it were not free.** | Statement that patient would NOT have enrolled in MITI if there was a cost. |
| **Desire for MITI to grow, expand, help other patients** |  | Statements that indicate the interviewee would like MITI to grow or expand to other health conditions. Also include statements that indicate interviewee would like MITI to help other patients. |
| **Patient motivations** | See below | This is a broad category capturing any statements about what motivates patients. Double-code other patient attitudes beliefs as necessary. For example, a patient might be motivated to enroll in MITI because regular diabetes management is a lot of work, so you can double code as ‘patient motivations’ and ‘diabetes management is expensive/burdensom.’ |
|  | **Patient motivated by health** | Statements that indicate that the patient is motivated by his/her own health. |
| **Unmet patient need for additional diabetes care or support** |  | Statements that indicate the interviewee perceives that there is an unmet patient need for diabetes care beyond MITI (e.g., education, support beyond insulin support). |
| **MITI is convenient/saves patient time/is easy to use** |  | Statements that indicate the interviewee believes that MITI is convenient for patients and/or saves patients time and/or is easy to use. |
|  | **MITI is a good alterantive to coming into the clinic** | Statements that indicate interviewee believes MITI is a good alternative to coming into the clinic. |
|  | **Technology (phone, text) makes MITI easy** | Statements that indicate the interviewee believes that the technological basis of MITI contributes to its ease of use, convenience, time-saving. |
| **Comfort or attitudes about sending texts to providers** | See below | Statements about patient comfort or attitudes about communicating via text with providers. Subcode valency (positive, negative, ambivalence) about texting providers. |
|  | **Patient comfortable, positive attidues about texting with providers** | Patient comfortable texting with providers |
|  | **Patient not comfortable, negative about attitudes texting with providers.** | Patient not comfortable texting with providers. |
| **Comfort or attitudes about MITI as an app or website** | See below |  |
|  | **Patient has positive attitudes about MITI as an app or website** | Statements indicating patient has positive attitudes about MITI being available as a phone app or website. |
|  | **Patient has negative attitudes about MITI as an app or website** | Statements indicating patient has negative attitudes about MITI being available as a phone app or website. |
|  | **Patient would prefer MITI as an app or website** | Statements indicating patient prefers MITI as an app or website (versus text) |
|  | **Patient prefers MITI as text (not app or website)/unable to use MITI as an app or website** | Statements indicating patient prefers MITI as a text program (versus app or website). Include statements that patient would not be able to use MITI if it were a website or app. |
|  | **Patient ambivalence or lack of preference about MITI as text, website or app** | Statements indicating patient is ambivalent or doesn’t have a preference about MITI being available in text, a phone app or website. |
| **Nurse concerns about scope of practice** |  | Statements indicating that nurses had concerns about how new responsibilities fit with scope of practice. Statements indicating nurses had concerns about new responsibilities in general. |
| **Chinese patients may not use text** |  | Statements indicating providers do not think Chinese-speaking patients use text messaging. |
| **Older, less tech-saavy patients may not use MITI** |  | Statements indicating providers do not think older or less tech-saavy patients will use MITI. |
